# Genetic variants of *FZD4* and *LRP5* genes in patients with advanced retinopathy of prematurity

**Published:** 2013-02-25

**Authors:** Hiroyuki Kondo, Shunji Kusaka, Aki Yoshinaga, Eiichi Uchio, Akihiko Tawara, Tomoko Tahira

**Affiliations:** 1 Department of Ophthalmology, University of Occupational and Environmental Health, Japan, Kitakyushu, Japan; 2 Department of Ophthalmology, Sakai Hospital, Kinki University Faculty of Medicine, Sakai, Japan; 3 Department of Ophthalmology, Osaka University Medical School, Osaka, Japan; 4 Division of Genome Analysis, Medical Institute of Bioregulation, Kyushu University, Fukuoka, Japan; 5 Department of Ophthalmology, Fukuoka University, Fukuoka, Japan

## Abstract

**Purpose:**

Retinopathy of prematurity (ROP) is a complex disease with a genetic predisposition, but little is known about its genetic background. It has a clinical resemblance to familial exudative vitreoretinopathy (FEVR), a hereditary disease characterized by defects in the development of retinal vessels. Several studies have suggested that mutations in the causative genes for FEVR may account for a proportion of advanced ROP, but conflicting data have also been reported for some variants. To address the possibility of genetic involvement of FEVR genes in ROP, we performed comprehensive sequence analyses of 53 Japanese patients with advanced ROP for the FEVR-causing genes.

**Methods:**

Peripheral blood DNA was obtained from 53 patients referred to our hospitals for retinal surgery. Polymerase chain reaction followed by direct sequencing of the coding regions of the known FEVR-causing genes (*FZD4, LRP5, TSPAN12,* and *NDP*) and a noncoding exon of the *NDP* gene was performed. Possible pathogenicity of the sequence changes were analyzed by orthologous protein sequence alignment and by computational predictions.

**Results:**

We identified six different nonsynonymous DNA variants in the coding region of either the *FZD4* gene (p.H69Y, p.R127H, and p.Y211H) or the *LRP5* gene (p.R1219H, p.H1383P, and p.T1540M) in seven patients. The corresponding codons of these changes were highly conserved among species, and these changes were predicted to be pathogenic by at least two of four computational prediction programs. No such changes were found in the *TSPAN12* and *NDP* genes.

**Conclusions:**

Six possibly pathogenic variants of *FZD4* or *LRP5* were found in seven advanced ROP patients. Although these variants do not yet provide definitive evidence that they are causal, the results imply a role of the *FZD4* and *LRP5* genes in the development of advanced ROP.

## Introduction

Retinopathy of prematurity (ROP) is a disorder affecting the development of the retinal vasculature in premature infants. ROP is a multifactorial disease, and many factors have been suggested to cause ROP, including low birth weight, young gestational age, and prolonged oxygen supplementation. In addition to such environmental factors, a strong genetic predisposition to ROP is suggested through recent research with the candidate gene approach, higher concordance rate in monozygotic twins, and other clinical and experimental animal studies [1]. Genetic variations of genes related to retinal vascular formation are considered to be associated with the development of advanced ROP; however, little is known about the exact genetic mechanism(s) [2,3].

The clinical characteristics of eyes with ROP are similar to those of familial exudative vitreoretinopathy (FEVR), a hereditary disorder affecting full-term infants. Both diseases share defects in the development of retinal vessels and secondary retinal pathologies, including retinal folds and retinal detachments. FEVR is a genetically heterogeneous disease, and mutations in the genes coding for the ligand–receptor complex of Wnt signaling in the retina (*FZD4*, *LRP5*, *TSPAN12,* and *NDP*) are known to cause FEVR [4-8].

Because of the phenotypic resemblance, genetic changes in FEVR-causing genes are considered to be involved in the progression of ROP. Several studies have addressed this possibility and have reported that variants of the *FZD4*, *LRP5,* and *NDP* genes account for 3% to 12% of the cases with ROP [1,9-14]. However, the types and incidences of these variants are different between reports, and some conflicting results have been reported in different ethnicities [1]. The functional importance and distinctions between mutations and polymorphisms have still not been determined in some of the variants [13,15]. Therefore, further studies are required to address the possibility of genetic involvement of the FEVR genes to ROP.

We performed a comprehensive sequence study of 53 Japanese cases with advanced ROP for FEVR-causing genes. We found six, rare, nonsynonymous, single nucleotide variants in *FZD4* or *LRP5* in seven patients, and we discussed their possible involvement in the etiology of ROP.

## Methods

### Participants and clinical examinations

Fifty-three patients with ROP who were referred to our hospitals for retinal surgery were studied. Our procedures conformed to the tenets of the Declaration of Helsinki. An informed consent was obtained from all parents. The experimental protocol was approved by the Ethics Review Board of the University of Occupational and Environmental Health, Fukuoka University, and Osaka University, Japan. All of the patients were Japanese and all were born at a gestational age of <30 weeks with a birth weight of <1,500 g. All patients received oxygen supplementation; however, details of the treatments were not available because they were born in other hospitals. The stage of ROP was determined according to the International Classification of Retinopathy of Prematurity [16].

### Laboratory studies

DNA samples were extracted from peripheral blood using a DNA extraction kit (QiaAmp; Qiagen, Chatsworth, CA). Prior to the extraction, the blood samples were mixed with EDTA and preserved in a freezer at −20 °C. To identify variants in all coding exons of the *FZD4*, *LRP5, TSPAN12,* and *NDP* genes, oligonucleotide primers on the flanking intron/untranslated region (UTR) sequences were designed and synthesized as described [17-19]. In addition, the noncoding exon 1 in the *NDP* gene was also tested.

Polymerase chain reaction (PCR) was performed with optimized annealing temperatures. The PCR products were treated with shrimp alkaline phosphatase (Roche Applied Science, Indianapolis, IN) and exonuclease I (NEB, Ipswich, MA), and then sequenced with the BigDye Terminator ver.1.1 (Applied Biosystems, Foster City, CA). The samples were denatured and analyzed with a DNA sequencer (3100 or 3730 Genetic Analyzer; Applied Biosystems). All sequence analyses were performed in both directions. Each sequence change was evaluated by two examiners using the Phred/Phrap/Consed program [20].

The allele frequency of each variant in the ethnicity-matched general population was estimated by referring to our previous reports [17-19,21]. These DNA samples were collected as a part of cohort study of cancer before 1995 and had been made anonymous. The use of these samples was approved by the Ethics Review Board of the Kyushu University. The retinal status of these samples was not determined.

### Bioinformatics analysis

The identified variants were examined to determine whether they were known polymorphisms with the allele frequencies by the dbSNP. Novel or known variants with <5% of allele frequency were evaluated further.

The FZD4 and LRP5 protein sequences of humans and other vertebrates were obtained from the UCSC Genome Browser and aligned against each other using the ClustalW software provided by EMBL-EBI, European Bioinformatics Institute [22]. The pathogenicity of the amino acid changes was determined by four computational programs; SIFT, Polyphen2, MutationTaster, and PhyloP [23-26]. PhyloP was retrieved from the dbNSFP database through the ANNOVAR annotation program [27,28]. Variants were considered to be pathogenic if they were predicted as “damaging (SIFT or Polyphen2),” “disease-causing (MutationTaster)” or “conserved (PhyloP)” by at least two of the programs. The reported *FZD4* variants were also re-evaluated by the programs.

## Results

Demographic data for the 53 infants are presented in Table 1. Twenty-eight infants were boys and 25 were girls. The average gestational age was 25.1 weeks with a range from 22 to 29 weeks, and the average birth weight was 769.2 g with a range from 420 to 1244 g. All of the eyes were at stage 4 or 5 except one patient who had both eyes at stage 3. All patients underwent vitrectomy or scleral buckling surgery in at least one eye except two eyes that were either inoperable or treated with intravitreal bevacizumab alone.

**Table 1:** Demographic data of 53 patients with advanced retinopathy of prematurity.

Gestational age	22 – 29 (mean 25.1) weeks
Birth weight	420 – 1,244 (mean 769.2) grams
Sex	Male: 28 (53%)
	Female: 25 (47%)
Type of ROP	Classic: 37 (70%)
	APROP: 13 (25%)
	Undetermined: 3 (6%)
Stage of ROP	Stage 5: 30 (57%)
	Stage 4B: 12 (23%)
	Stage 4A: 10 (19%)
	Stage 3: 1 (2%)

Type and stage of retinopathy of prematurity (ROP) were based on the International Classification of Retinopathy of Prematurity [16]. Stage of ROP is shown for the more severely affected eye.

### Novel or known variants detected in the *FZD4* gene

We identified four different single-base substitutions in the coding sequence of the *FZD4* gene in five patients. All variants were heterozygous changes. Three variants were nonsynonymous changes (Table 2): c.205C>T (p.H69Y), c.380G>A (p.R127H), and c.631T>C (p.Y211H). The other was a novel synonymous change c.696C>T (p.I232I), which was predicted to be “tolerated (SIFT)” and “polymorphism (Mutation Taster),” and was excluded from the following screening.

**Table 2:** Rare *FZD4* variants identified in patients with advanced retinopathy of prematurity

Location	cDNA change	Protein change (score with Blosum62)	Occurrence in patients	Allele frequency in ethnically matched samples	Known polymorphism (rs ID, allele frequency)	Computational analysis score				Patient characteristics: ethnicity; gender; birthweight; gestational age; clinical data	Reference
						SIFT	Poly- phen2	Muta- tion Taster	Phylop		
Exon 1	c.205C>T	p.H69Y (2)	1/53	2/300	Yes (rs80358282)	**0.02 (D)**	*0.197 (B)*	**0.9907 (Dc)**	**0.9992 (C)**	#N5301: Japanese; male; 460 g; 24 gw; APROP/stage4A (OU), Lx at 2 months (OU), Vx at 4 months (OU)	This study
Exon 2	c.380G>A	p.R127H (0)	1/53	0/300	Yes (rs184709254 0.05%)	*0.11 (T)*	**0.984 (PrD)**	**0.9968 (Dc)**	**0.9818 (C)**	#N3401: Japanese; male; 801 g; 25 gw; APROP/stage 5 (OU), Lx at 1 month, Vx (OU)	This study
Exon 2	c.502C>T	p.P168S (−1)	7/71 [15] 4/60 [37]	12/346 [15] ND [37]	Yes (rs61735303, 1.424%)	**0.02 (D)**	*0.146 (B)*	**0.9990 (Dc)**	**0.9997 (C)**	ND	[15,37]
Exon 2	c.609G>T	p.K203N (0)	1/71	0/346	No	*0.13 (T)*	*0.415 (B)*	**0.9913 (Dc)**	**0.9841 (C)**	Caucasian; male; 780 g; 26 gw; Stage 3 (OU), Lx at 78 d	[15]
Exon 2	c.631T>C	p.Y211H (2)	2/53	0/300	No	**0.02 (D)**	*0.003 (B)*	**0.9983 (Dc)**	**0.9818 (C)**	#N1901: Japanese; female; 1,152 g; 27 gw; Classic/stage 5 (OU), Vx (OD), glaucoma (OS), #N4001: Japanese; male; 566 g; 23 w; APROP/stage 4A (OU), Lx & Vx (OU)	This study
Exon 2	c.766A>G	p.I256V (3)	1/20	0/200	Yes (rs10489423, 0.179%)	*0.11 (T)*	**0.938 (PsD)**	**0.9900 (Dc)**	**0.9981 (C)**	ND	[36]
Exon 2	c.1109C>G	p.A370G (0)	1/71	0/346	No	*1 (T);*	*0.058 (B)*	**0.9987 (Dc)**	**0.9997 (C)**	Caucasian; male; 880 g; 28 gw; Stage 3 (OU), Lx at 92 d	[15]
Exon 2	c.1396C>T	p.R466W (−3)	1/71	0/346	No	**0.01 (D)**	**0.999 (PrD)**	**0.9949 (Dc)**	**0.9705 (C)**	Mixed (Chinese and Caucasian); female; 650 g; 24 gw; Stage 3 (OU), Lx at 68 d	[15]

Allele frequency from ethnically-matched samples was obtained from either random or control individuals. Known polymorphism was based on the dbSNP, and the allele frequency was on the Exome Variant Server, NHLBI Exome Sequencing Project (ESP), Seattle, WA. Computational analyses were performed by 4 programs (SIFT, Polyphen2, Mutation taster, and PhyloP). Pathogenic results were shown by **bold** characters as “D (damaging)” for SIFT, “PrD (probably damaging)” or “PsD (possibly damaging)” for Polyphen2, “Dc (disease-causing)” for Mutation Taster, and “C (conserved)” for PhyloP. Non-pathogenic results are shown in *italics* as “T (tolerated)” for SIFT, and “B (benign)” for Polyphen2. Note p.P168S was found as a compound mutation with p.P33S, however, p.P33S was not listed because this study showed it was “non-pathogenic” by SIFT, Polyphen2 and Mutation Taster. d: days after birth, g: gram, gw: gestational weeks, Lx: laser, m: months after birth, ND: not described, Vx: vitrectomy, w: weeks after birth.

The clinical manifestations of the four patients who carried the nonsynonymous change are shown in Table 2. The p.H69Y change was found in one boy with aggressive posterior (AP) ROP (#N5301). He was born at a gestational age of 24 weeks with a birthweight of 460 g. Bilateral laser photocoagulation was performed at the conceptual age of 33 weeks and vitrectomy at 42 conceptual weeks. The p.R127H variant was found in a boy with AP-ROP (#N3401). This child was born at a gestational age of 25 weeks with a birth weight of 801 g. The retinopathy progressed aggressively, and he required laser treatment at 32 post-conceptual weeks; however, the retinopathy progressed to total retinal detachment in both eyes. Lensectomy and vitrectomy were performed on both eyes, but the retina could not be reattached.

The variant p.Y211H was found in two patients. One patient was a girl with classic ROP (#N1901) who was born at a gestational age of 27 weeks with a birthweight of 1,152 g. The retinopathy progressed to stage 5 in both eyes. Vitrectomy was performed on the right eye but not on the left eye because of corneal opacity and glaucoma. The other patient was a boy with AP-ROP (#N4001) who was born at a gestational age of 23 weeks with a birth weight of 566 g. The retinopathy progressed to stage 4A ROP bilaterally, and early vitrectomy was performed following laser therapy on both eyes.

**Figure 1:**
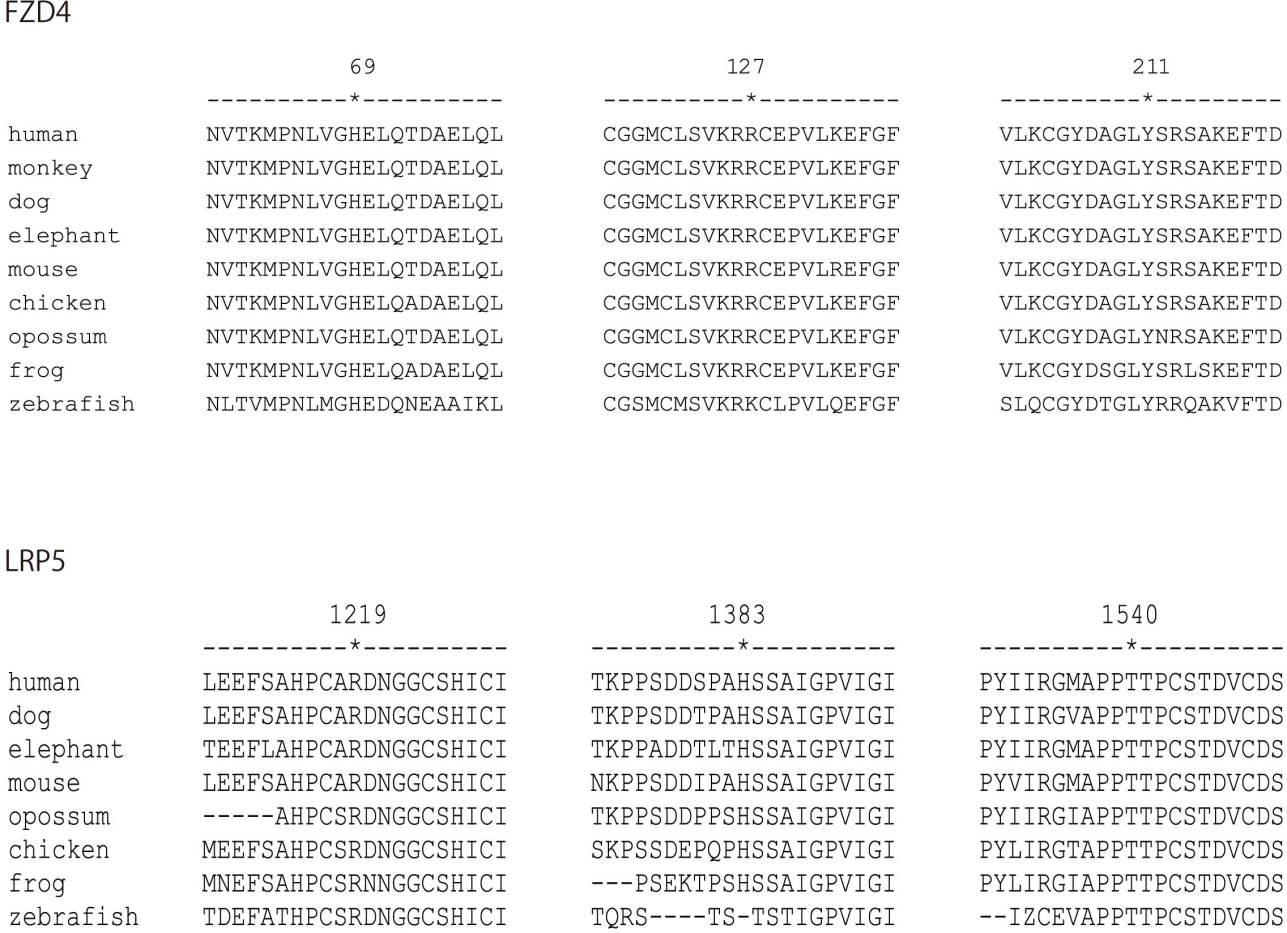
Sequence alignment of the FZD4 and LRP5 proteins. The FZD4 (upper panel) and LRP5 (lower panel) protein sequences of humans and other vertebrates were obtained from the UCSC Genome Browser and aligned against each other using the ClustalW software provided by EMBL-EBI, European Bioinformatics Institute [22]. Amino Acids are shown by one letter code and asterisks indicate the corresponding codons to nonsynonymous variants found in this study. Codons H69, R127 and Y211 of FZD4, and codons R1219, H1383 and T1540 of LRP5 are highly conserved in vertebrates.

Codon 69 and 127 were located in the cysteine-rich domain of the extracellular region of the *FZD4* gene. Codon 211 is located at the Frizzled/Smoothened family membrane region but outside the cysteine-rich domain. Codon 69, 127, and 211 are highly conserved in vertebrates (Figure 1). These amino acid substitutions were predicted to be pathogenic by at least two of the four programs (Table 2).

The p.H69Y change was already known to be present in patients with FEVR [17,29,30]. This change was assigned to rs80358282 as an OMIM disease-associated allele. The allele frequency was reported to be 0.046% (2/4338) according to the Exome Variant Server, NHLBI Exome Sequencing Project (ESP), Seattle, WA (accessed July 2012), and 1.69% in the 1000 Genome project (Japanese population). Our earlier study found 2/300 alleles (0.6%) from 150 samples in the Japanese population [17]. The p.R127H change was not found in the same 150 samples but was found in the dbSNP database (build 135) assigned to rs184709254. The minor allele frequency was estimated to be 0.05% in the 1000 Genome project. The p.Y211H change was not found either in 150 DNA samples or in the dbSNP database (build 135, [accessed July 2012]).

The retinal features and laboratory data of family members were only available for two individuals. The father of patient #N5301 carried the p.H69Y change and no sign of FEVR was found. The mother of patient #N4001 did not carry the p.Y211H change and had no ocular symptoms or family history of FEVR but had minimal vascular changes without retinal avascularization in the eyes and had macular macrovessels in the left eye.

### Novel or known variants detected in *LRP5*

We identified ten different single-base substitutions in the coding sequence in the *LRP5* gene. Of the ten variants, five were synonymous changes and three were known variants: c.2220C>T (p.N740N, rs2306862), c.3357A>G (p.V1119V, rs556442), and c.4089C>T (p.D1363D, rs3736229). These synonymous changes were common polymorphisms of >10% of minor allele frequency. Two were novel variants: c.285G>A (p.T95T) and c.3156G>A (p.R1052R). These synonymous changes were predicted as “polymorphism” by MutationTaster and were excluded from the following screening.

The other five variants were nonsynonymous changes. Two were common polymorphisms: c.266A>G (p.Q89R, rs41494349) and c.3989C>T (p.A1330V, rs3736228). These variants were excluded from the following screening.

The remaining three variants were found in three patients, and we searched for their pathogenicity (Table 3, Figure 1). All variants were heterozygous changes. Two were rare polymorphisms: one was c.3656G>A (p.R1219H, rs143924910), and the allele frequency was reported to be 0.022% in the Caucasian population according to NHLBI-ESP. The other was c.4619C>T (p.T1540M, rs141407040), and the allele frequency was reported to be 0.022% in NHLBI-ESP and 1.69% in Japanese in the 1000 Genome project. Our earlier study showed that the frequency was 4/386 alleles (1.04%) from 193 DNA samples in the Japanese population [21]. This change was found in patients with FEVR but was not regarded as a mutation [21].

**Table 3:** Rare *LRP5* variants identified in advanced retinopathy of prematurity patients

Location	cDNA change	Protein change (score with Blosum62)	Occurrence in patients	Allele frequency in ethnically matched samples	Known polymorphism (rs ID, allele frequency)	Computational analysis score				Patient characteristics: ethnicity; gender; birthweight; gestational age; clinical data	Reference
						SIFT	Poly- phen2	Mutation Taster	Phylop		
Exon 17	c.3656G>A	p.R1219H (0)	1/53	NA	Yes (rs143924910, 0.022%)	*0.59 (T)*	**0.996 (PrD)**	**0.9988 (Dc)**	**0.9904 (C)**	#N5701: Japanese; male; 864 g; 26 gw; Classic ROP, Stage 4A (OU), Lx at 8 w, Vx at 13 w	This study
Exon 20	c.4148A>C	p.H1383P (−2)	1/53	1/386	No	*0.26 (T)*	*0.001 (B)*	**0.9983 (Dc)**	**0.9975 (C)**	#N1701: Japanese; female; 719 g; 25 gw; Classic ROP, Stage5 (OD), Stage 4B (OS), Lx (OU), Vx at 6 m (OD) and 4 y (OS)	This study
Exon 23	c.4619C>T	p.T1540M (−1)	1/53	4/386	Yes (rs141407040, 0.022%)	**0 (D)**	**1.00 (Prd)**	**0.9908 (Dc)**	**0.9617 (C)**	#N1001: Japanese; male; 850 g; 25 gw; Classic ROP, Stage 4B (OS) / 5(OD), Lx (OU), Vx at 6 m (OU)	This study

Allele frequency from ethnically-matched samples was obtained from random individuals. Known polymorphism was based on the dbSNP, and the allele frequency was on the Exome Variant Server, NHLBI Exome Sequencing Project (ESP), Seattle, WA. Computational analyses were performed by 4 programs (SIFT, Polyphen2, Mutation taster, and PhyloP). Pathogenic results are shown by bold characters as “D (damaging)” for SIFT, “PrD (probably damaging)” or “PsD (possibly damaging)” for Polyphen2, “Dc (disease-causing)” for Mutation Taster, and “C (conserved)” for PhyloP. Non-pathogenic results are shown by *italic* characters as “T (tolerated)” for SIFT, and “B (benign)” for Polyphen2. g: gram, gw: gestational weeks, Lx: laser, m: months after birth, NA: not analyzed, Vx: vitrectomy, y: years after birth.

The last variant was c.4148A>C (p.H1383P) and it was not found in the dbSNP database, but our earlier study showed the frequency was 1/386 alleles (0.26%) from 193 DNA samples in the Japanese population.

The clinical manifestations of the three patients who carried the nonsynonymous changes are shown in Table 3. The p.R1219H change was found in a male child with classic ROP (#N5701). He was born at a gestational age of 26 weeks with a birth weight of 864 g. Despite photocoagulation at 34 conceptual weeks, both eyes progressed to stage 4A, and vitrectomy was performed on both eyes at 39 conceptual weeks. The p.H1383P change was also found in a female child with classic ROP (#N1701). She was born at a gestational age of 25 weeks with a birth weight of 719 g. Despite photocoagulation, retinopathy progressed to stage 5 (OD) and stage 4B (OS). Vitrectomy was performed on the right eye at 6 months of age and on the left eye at 4 years as a late recurrent retinal detachment.

The p.T1540M change was found in one boy with classic ROP (#N1001). He was born at a gestational age of 25 weeks with a birth weight of 850 g. Despite photocoagulation, the retinopathy progressed to stage 5 (OD) and stage 4B (OS). Vitrectomy was performed on both eyes at 6 months of age. The retinal features and laboratory data of family members were unavailable for these patients.

Codon 1219 is located between the fourth beta-propeller domain and the low density lipoprotein receptor domain of LRP5. Codon 1383 is adjacent to the transmembrane domain of LRP5. Codon 1540 is located in the second PPPSP motif in the intracellular domain of LRP5. These amino acid substitutions are highly conserved in vertebrates (Figure 1) and were predicted to be pathogenic in at least two of the four programs (Table 3).

We also found three or six base nucleotide insertions at the repeat sequence of exon 1 in three patients (insertions of CTG for #N2501 and #N5501 and an insertion of CTG CTG for #N201). These were predicted to elongate the leucine repeat changes and were found to be at a known polymorphic repeat site (rs72555376) [31]. Because these were common polymorphisms, we could not assess the pathogenicity further.

No sequence changes were identified for *TSPAN12* and *NDP* except two common single nucleotide polymorphisms in *TSPAN12* (rs41623 and rs41624). Both changes are known to have a minor allele frequency of >10% and are likely not to be pathogenic.

## Discussion

Our findings showed that seven of 53 advanced ROP patients carried six novel or rare nonsynonymous variants either in the *FZD4* or the *LRP5* gene. The corresponding codons for these changes were highly conserved and predicted to be pathogenic by computational analyses. No such sequence changes were identified in the *TSPAN12* and *NDP* genes. To the best of our knowledge, this is the first study to perform genetic screening of all known FEVR-causing genes in a cohort of patients with advanced ROP.

### Heterogeneous DNA changes found in advanced retinopathy of prematurity

Different kinds of DNA changes have been found in three genes (*FZD4*, *LRP5,* and *NDP)* in patients with advanced ROP. These are common or rare (novel) changes, and the variants were located in the UTRs or coding regions. These variants are highly heterogeneous so the relevance to biologic significance needs to be evaluated carefully.

Common variants can be tested for their significance by association studies under the assumption of the disease-common variant hypothesis [32]. Haider et al. identified a polymorphism in 5′UTR of the *NDP* gene (C597A) that was associated with severe ROP in a Kuwaiti population [12]. However, the pathogenicity of the substitution is unclear, and no other study has addressed the association in different ethnic populations.

Hiraoka et al. identified a three-base (CTG) insertion in the coding region of the *LRP5* gene [14] in one of 17 samples, and we also found the identical variant and a six-base (CTG CTG) insertion in the same position of the *LRP5* gene. These changes were thought to be commonly found as polymorphisms, but no association study has been performed. Although these changes were predicted to elongate the leucine repeat in the signal sequence, suggesting a pathogenic character [31], the significance of the association with ROP needs to be evaluated further in a larger number of cohorts.

The other types of variants are rare or novel variants. These variants are likely to be of fairly recent origin and are not suitable for association studies because their rarity makes it difficult to obtain statistical significance. As an alternative to the disease-common variant hypothesis, the mutation-selection hypothesis proposes that much of the susceptibility is due to rare variants [32]. We believe that some known rare variants are as important as novel mutations for pathogenicity, and these should be evaluated together for ROP. The dbSNP database (build 135) contains >53×10^6^ human variations and includes not only common benign polymorphisms but clinically associated variants. In addition, some rare variants are newly identified to be the cause of Mendelian diseases. For example, p.R207W in the *NMNAT1* gene (rs150726175), which was reported to be 0.098% of minor allele frequency, was found to be responsible for ocular macular atrophy [33].

There are two different types of rare variants, variants in the UTRs and missense variants (nonsynonymous) in the coding regions. The putative disease-associated variants located in the UTRs are only found in the *NDP* gene [10-14,34]. These are insertions, deletions, and single-base substitutions either in the 5′ or 3′ UTR. These regions play a role of gene regulation, and variants in the 5′ UTR of the *NDP* gene have been evaluated by a functional analysis [35]. However, variants in the 3′ UTR have not been functionally evaluated and their significance remains unknown. Recently, Hiraoka et al. screened 17 Japanese patients with advanced ROP [14] and identified a heterozygous substitution in the 5′ UTR of the *NDP* gene. We examined the 5′UTR of the *NDP* gene but did not detect any genetic variants. Such rare variants account for only a small fraction of patients with ROP, thus it is not surprising that different screening studies have identified different variants even in the same ethnic population.

The other type of rare or novel variants is the DNA substitutions in the coding regions that have been found as nonsynonymous variants either in the *NDP* or *FZD4* gene. It is difficult to distinguish between benign amino acid substitutions from mutant amino acid substitutions that cause a disruption of the protein structure and/or an impairment of function. Computational analyses have been used for the first screening but they usually do not provide definitive evidence as different programs provide different assessment of pathogenicity. Although further evaluations are required for pathogenicity, these programs are useful to exclude benign variants.

Four different changes in the *FZD4* gene, viz., p.K203N, p.I256V, p.A370G, and p.R466W, were uniquely found in patients with advanced ROP [15,36], while two mutations in the *NDP* gene, viz., p.L108P and p.R121W [9], and one compound mutation in the *FZD4* gene, viz., p.P33S/p.P168S [15,37], were found either in patients with advanced ROP (Table 2) or FEVR. We found three variants of *FZD4,* one of which is novel and the others are known variants with <1% of allele frequency. Two variants were unique to ROP (p.R127H and p.Y211H), and the other (p.H69Y) was found in FEVR patients [17,29,30]. In addition, we identified three nonsynonymous variants (p.R1219H, p.H1383P, and p.T1540M) of the *LRP5* gene that were variants with <2% of allele frequency. These variants have not been reported to be FEVR causing, but the p.T1540M variant was found in FEVR patients without concordant segregation [21].

### Phenotype–genotype correlation for familial exudative vitreoretinopathy and retinopathy of prematurity

The Wnt signaling genes control the development of the retinal vasculature, and impairments of Wnt signaling by mutations can cause defective vascular growth. This leads to retinal hypovascularization, which is the predominant feature of FVER and ROP [1]. Along with prematurity and other systemic abnormalities, the retinopathy in patients carrying these genetic mutations may tend to be exacerbated. It is known that the severity of the mutations of the Wnt signaling genes causes different phenotypes for FEVR and the allied inherited diseases. For example, mutation in *NDP* causes either FEVR or Norrie disease (a more severe phenotype), and mutation in *LRP5* causes FEVR or osteoporosis pseudoglioma syndrome, a more severe phenotype [4,6]. Phenotypic severities are related to the severity of the mutational effects [18,21]. Therefore, we hypothesize that advanced ROP is related to milder functional impairments of the Wnt genes, while FEVR and the allied disorders are caused by more severe impairments of the genes.

In support of this hypothesis, a distinct mutational spectrum has been proposed for *FZD4* between FEVR and ROP [15]. FEVR-causing mutations are located in important functional areas, i.e., the cysteine-rich domain, transmembrane domains, cytoplasmic domains, and C-terminal tail [15]. Contrary to FEVR, previously reported variants of *FZD4* that are unique to ROP, viz., p.K203N, p.I256V, p.A370G, and p.R466W, tend to be milder nucleotide substitutions or are located in less important regions [15,36,37]. Our results agree with this hypothesis. Thus, Y221 is located outside the cysteine-rich domain. R127 and H69 are located in the cysteine-rich domain; however, the corresponding amino acid substitutions either retain the polar property of the protein or provide less severe substitution by the blosum62 matrix (Table 2). A similar distinct spectrum remains to be determined for the *LRP5* gene.

There are variants that cause intermediate severity as found in patients with either ROP or FEVR. We found an p.H69Y change in *FZD4* that has been reported as FEVR causing. FEVR patients with p.H69Y often show mild or no retinal changes, which has been considered to be due to low penetrance [17]. In addition, p.H69Y was found in several patients as a second mutation accompanying other FEVR mutations, suggesting a role as a phenotypic modifier [17,29]. A similar clinical manifestation of low penetrance was reported for the p.P33S/p.P168S change [36,38]. Thus, we suggest that variants of intermediate severity underlie some patients with ROP or FEVR, the latter manifest complex genetic traits rather than a simple monogenic inheritance.

### Functional significance

We have performed in vitro assays to determine the effects of FEVR-associated single nucleotide variants in the *FZD4*, *LRP5,* and *NDP* genes systematically [39]. The results demonstrated that Wnt signal transduction was completely stopped by a nonsense mutation in the *FZD4* gene, while the transduction was moderately reduced (26% to 48%) by nonsynonymous variants (missense mutations) of either *FZD4, NDP,* or *LRP5*. In addition, some known polymorphisms of *FZD4* and *LRP*, including p.H69Y in *FZD4* and p.T1540M in *LRP5,* were shown to lead to milder but significant reductions in signal transduction [39]. The assays provided evidence of the functional impairments caused by the variants, and the data are concordant with the milder phenotypes of the patients who carry them.

In conclusion, we found six rare nonsynonymous single nucleotide variants in seven patients. These were computationally predicted as pathogenic variants, although the findings on these variants do not yet provide definitive evidence that they are causal. Our results imply the role of the *FZD4* and *LRP5* genes in the development of advanced ROP. A weakness of this study is four of six variants have not been evaluated by functional assays. Association studies are yet to be performed, especially for variants such as the leucine repeat of *LRP5*. We cannot assess whether variants found in this study are de novo or inherited, and with a few exceptions. the fundus appearances of the relatives were not obtained.

Currently, mutations in the *FZD4*, *LRP5*, *TSPAN12,* and *NDP* genes have not been found in nearly one-half of patients with FEVR. This would suggest that other FEVR-related genes possibly related to Wnt signaling are present [7,40]. Further studies examining unknown Wnt-signaling genes should provide information for the better understanding of the pathophysiology of ROP.
